# A Statistical Parametric Mapping Toolbox Used for Voxel-Wise Analysis of FDG-PET Images of Rat Brain

**DOI:** 10.1371/journal.pone.0108295

**Published:** 2014-09-26

**Authors:** Binbin Nie, Hua Liu, Kewei Chen, Xiaofeng Jiang, Baoci Shan

**Affiliations:** 1 Key Laboratory of Nuclear Analysis Techniques, Beijing Engineering Research Center of Radiographic Techniques and Equipment, Institute of High Energy Physics, Chinese Academy of Sciences, Beijing, China; 2 Banner Alzheimer’s Institute, Banner Good Samaritan Positron Emission Tomography Center, Phoenix, Arizona, United States of America; 3 Department of Mathematics and Statistics, Arizona State University, Tempe, Arizona, United States of America; 4 Department of Radiology, University of Arizona, Tucson, Arizona, United States of America; 5 Arizona Alzheimer’s Consortium, Phoenix, Arizona, United States of America; 6 School of Public Health and Family Medicine, Capital Medical University, Beijing, China; Glasgow University, United Kingdom

## Abstract

**Purpose:**

PET (positron emission tomography) imaging researches of functional metabolism using fluorodeoxyglucose (^18^F-FDG) of animal brain are important in neuroscience studies. FDG-PET imaging studies are often performed on groups of rats, so it is desirable to establish an objective voxel-based statistical methodology for group data analysis.

**Material and Methods:**

This study establishes a statistical parametric mapping (SPM) toolbox (plug-ins) named spmratIHEP for voxel-wise analysis of FDG-PET images of rat brain, in which an FDG-PET template and an intracranial mask image of rat brain in Paxinos & Watson space were constructed, and the default settings were modified according to features of rat brain. Compared to previous studies, our constructed rat brain template comprises not only the cerebrum and cerebellum, but also the whole olfactory bulb which made the later cognitive studies much more exhaustive. And with an intracranial mask image in the template space, the brain tissues of individuals could be extracted automatically. Moreover, an atlas space is used for anatomically labeling the functional findings in the Paxinos & Watson space. In order to standardize the template image with the atlas accurately, a synthetic FDG-PET image with six main anatomy structures is constructed from the atlas, which performs as a target image in the co-registration.

**Results:**

The spatial normalization procedure is evaluated, by which the individual rat brain images could be standardized into the Paxinos & Watson space successfully and the intracranial tissues could also be extracted accurately. The practical usability of this toolbox is evaluated using FDG-PET functional images from rats with left side middle cerebral artery occlusion (MCAO) in comparison to normal control rats. And the two-sample t-test statistical result is almost related to the left side MCA.

**Conclusion:**

We established a toolbox of SPM8 named spmratIHEP for voxel-wise analysis of FDG-PET images of rat brain.

## Introduction

PET (positron emission tomography) imaging techniques of functional metabolism using fluorodeoxyglucose (^18^F-FDG) have been increasingly used for the investigation of human brain functions in normal and diseased individuals [1]–[3]. Complementary to human brain studies, animal experiments are important for pathogenesis research, therapeutic efficacy evaluation, and drug development, for it allows transverse comparisons between different brain regions or between different rats, and longitudinal follow-up. While numbers of small-animal FDG-PET imaging studies are performed on groups of rodents, how to analyze images for efficient interpretation of physiological significance is a pivotal problem to be solved. In ^18^F-FDG imaging studies of human brain, the statistical parametric mapping (SPM, Wellcome Department of Cognitive Neurology, London, UK) is one of the most popular software for image analysis voxel-by-voxel. Groups of human brain images can be statistical analyzed in SPM objectively and automatically, which is also desirable in rodent studies.

In order to eliminate individual differences in voxel-wise analysis, the spatial normalization should be performed primarily, in which a standard template is the reference target image. Because of the relatively large difference in spatial resolution between MRI (magnetic resonance imaging) and FDG-PET images of small animals, automated image co-registration of FDG-PET data with MRI templates is difficult. Therefore, the established MRI template [4]–[6] could not be used in FDG-PET studies of rat brain. Casteels et al. [7] has first constructed an FDG-PET template of rat brain in Paxinos & Watson space [8] used for spatial normalization. The introduction of their template has accelerated a number of FDG-PET studies in rats [9], [10]. However, we noted that this template do not include the olfactory bulb of rat. As implied in some important researches recently, there is a close association between olfactory dysfunction and cognitive impairment [11]–[16]. Therefore, the olfactory bulb is necessary not only in olfaction study but also in cognitive study, which should be included in template image of rat brain. In addition, in Casteels’ study [7], only ten female rats ranged from 13 to 34 weeks old were used for the template construction. Hence, in order to improve the group representation of the template for both male and female rats, and to better account for the brain variability due to aging process, our current work will include rats with more balanced male/female ratio (twelve male and twelve female adult rats) and narrower age range (10∼13 wk old).

The extracranial tissues were unwanted in imaging data analysis. Recently, the intracranial tissues of each rat are usually traced out manually [5], [7], which is subjective, labor-intensive and time-consuming. In the human, the extracranial tissues could be automatically removed by an intracranial mask after spatial normalization, of which idea could also be used in rat brain studies. Therefore, in this study, an intracranial mask corresponding to our constructed FDG-PET template will be established to remove extracranial tissues of individual images automatically after spatial normalization.

Furthermore, the assignment of anatomical location to functional effects is critical in the interpretation physiological significance of statistical result of voxel-wise analysis. For anatomical localization, an atlas space is prerequisite, such as the Tailarach space for human brain studies [17]. In rat brain studies, the stereotaxic coordinates by Paxinos & Watson [18] is one of the most widely used. Previously, several groups established digital coordinates [5], [7] or atlas image [4], [6] for anatomical localization. However, only a digital atlas is not enough for functional location. It is prerequisite to standardize the template into the atlas space [5], [7]. When the FDG-PET template is fitted into the Paxinos & Watson space, functional effects could be precisely allocated to anatomical structures, which could also be used to define the uptake in the structures [10].

To align the template into the atlas space, Schweinhardt et al. [5] used the landmark-based linear registration techniques with forty-nine homologous anatomical landmarks, which performed successfully in structural MRI template. In FDG-PET studies of rat brain, Rubins et al. [19] registered FDG-PET images to a synthetic FDG-PET target image which was constructed from atlas-derived VOI (voxel of interest) images using rigid body transformation, which has only six anatomical regions. Casteels et al. [7] standardized the functional template into the Paxinos space via the MRI template [5] using linear transformation method based on the mutual information maximization algorithm in SPM2. However, because the template image and the atlas are constructed from different subjects, only linear transformation is not sufficient to standardize the template into the atlas space. In order to combine both linear and non-linear transformation method in co-registration of the template and the atlas, Coello et al. [10] constructed synthetic FDG-PET image from MRI image, by segmenting the MRI image into white matter (WM) and gray matter (GM) and then conducting a weighted sum of the GM and WM maps. Thus, an individual FDG-PET image can be standardized into the Paxinos & Watson space while it was spatially normalized to the synthetic FDG-PET template. However, the spatial resolution of the synthetic FDG-PET image with only two resolvable regions is too low. Therefore, in our study, in order to improve the co-registration accuracy, six main anatomy structures, all of which could be identified in the FDG-PET template according to the voxel intensity ranges and locations, will be defined from an atlas image [4] in Paxinos & Watson space [18] to construct a synthetic FDG-PET image. Then, both linear and non-linear transformation method will be employed to standardize the FDG-PET canonical brain into the Paxinos & Watson space [18] via the synthetic FDG-PET image.

With the introduction above, the current study is designed to establish a voxel-wise analysis method of FDG-PET images of rat brain. In detail, an FDG-PET rat brain template, comprised cerebrum, cerebellum, olfactory bulb and extracranial tissues, will be constructed which is used for spatial normalization of individual rat brain images. An intracranial mask image in the FDG-PET template space will be constructed for extracranial tissues automatic removing. The FDG-PET template will be standardized into the Paxinos & Watson space for the localization of functional investigation. Finally, the constructed template sets are compiled as a SPM8 toolbox (plug-ins) named spmratIHEP. Moreover, to evaluate the usefulness of the toolbox implemented in SPM8, we analyze the data from rats with left side middle cerebral artery occlusion (MCAO) and those without. The spmratIHEP toolbox is available by contacting the corresponding author at shanbc@ihep.ac.cn.

## Materials and Methods

### 2.1. Animals and data acquisition

Twenty-four Sprague-Dawley (SD) rats of either sex, 10∼13 weeks old, weight 350 g±20 g, were used for template construction. A further ten similar rats were used for evaluation of spatial normalization and removal of extracranial tissues. Voxel-wise analysis of brain images was done on male SD rats, 9∼11 weeks old; weight 300 g±20 g; 9 underwent left side middle cerebral artery occlusion (MCAO) and 8 were healthy controls. Intraluminal occlusion of the middle cerebral artery (MCA) was accomplished using a modification of the Longa technique [20]–[22]. Under an operating microscope, the left common carotid artery (CCA), external carotid artery (ECA), and internal carotid artery (ICA) were exposed through a midline incision. The vagus nerve was carefully preserved as far as possible. After proximal CCA was ligated, a 3-0 uncoated monofilament nylon suture with rounded tip (2634,Beijing Sunbio Biotech Co., Ltd. China) was inserted from the lumen of the distal CCA and advanced into the ICA, approximately 18±0.5 mm beyond the bifurcation until mild resistance indicated that the tip was lodged in the anterior resistance cerebral artery, thus blocking blood flow to the middle cerebral artery. All the rats were deprived of food for 12–15 h before ^18^F-FDG injection, but had access to drinking water at all time [23].

^18^F-FDG was prepared at PET center of China PLA General Hospital. For each rat, ^18^F-FDG (18.5 MBq/100 g of body weight) was administered via tailvein injection without anesthesia. Then the rats were kept in their cages and placed in a room with minimal ambient noise for the ^18^F-FDG uptake. The uptake period was 40 min for maximization of ^18^F-FDG uptake in the brain [24]. Then the rats were anesthetized with isoflurane inhalation anesthesia (2% in 100% oxygen; IsoFlo: Hebei Jiumu Phama, ltd, China) using a nose cone.

For the twenty-four rats used in template construction, FDG-PET imaging was performed at the PET center of China PLA General Hospital in MicroPET/CT imaging system (eXplore Vista-CT, GE, USA), of which the radial spatial resolution is 1.0 mm full-width at half-maximum (FWHM) at the centre of the field of view (FOV). During FDG-PET scan, all the rats were anesthetized with isoflurane (the same as described above) and placed in the scanner with a plastic stereotactic head holder in prone position on the scanner bed. And the rat brain was centered in the FOV to perform a static acquisition of 10 minutes. Images were subsequently reconstructed using 3D ordered set expectation maximization (3D-OSEM) algorithm. Corrections for dead time, decay, attenuation, random coincidences and scattering were applied. Images were reconstructed on a 175×175×61 matrix, where the voxel size equals 0.39×0.39×0.77 mm. All scans were saved as Analyze format.

For the ten rats used for evaluation of spatial normalization and removal of extracranial tissues by the spmratIHEP toolbox, FDG-PET images were acquired on a micro PET system (E-plus166, Institute of High Energy Physics, CAS, China), of which the radial spatial resolution is 1.67 mm FWHM at the centre of the FOV. During FDG-PET scan, all the rats were anesthetized with isoflurane (the same as described above) and placed in the scanner with a plastic stereotactic head holder in prone position on the scanner bed. And the rat brain was centered in the FOV to perform a static acquisition of 30 minutes. Images were subsequently reconstructed using Filtered Back projection (FBP) algorithm. Images were reconstructed on a 128×128×63 matrix, where the voxel size equals 0.5×0.5×1 mm. All scans were saved as Analyze format.

The nine rats with MCAO and eight ones without were used for evaluation of this toolbox spmratIHEP for its capacity for functional localization. Twenty-four hours after operation, FDG-PET images were acquired on Siemens Inveon PET (Siemens Medical Solutions), of which the radial spatial resolution is 1.4 mm FWHM at the centre of the FOV. During FDG-PET scan, all the rats were anesthetized with isoflurane (the same as described above) and placed in the scanner with a plastic stereotactic head holder in prone position on the scanner bed. And the rat brain was centered in the FOV to perform a static acquisition of 20 minutes. Images were subsequently reconstructed using FBP algorithm. Corrections for dead time, decay, attenuation, random coincidences and scattering were applied. Images were reconstructed on a 128×128×159 matrix, where the voxel size equals 1.4×1.4×0.79 mm. All scans were saved as Analyze format.

All experiments were performed with the approval of the Animal Care and Use Committee of the Chinese Academy of Sciences and conformed to named international guidelines on the ethical use of animals.

### 2.2. Template construction

#### 2.2.1. Construction of an average template image

The FDG-PET rat brain template image with the extracranial tissues was created using SPM8. All of the 24 images included in this study were inspected and were found equally of high quality in terms of the image contrast, noise level, and resolution. Firstly, the voxel size of all the brain images of 24 rats were adjusted to [1 1 1.8], which was scaled up in the Analyze header by the factor of 4 to better approximate human dimensions [5]. Then, the images were manually sheared to roughly remove voxels of the body and background by MRIcro [25]. Afterwards, the average template image was created recursively by registering [26]–[28] and averaging. In detail, one of these 24 rat brains was selected as the initial brain template. Each rat brain image was spatially normalized to this initial rat brain template, using the affine transformation and subsequent non-linear warping algorithm [29] implemented in SPM8.

The 24 spatially normalized images were averaged to create a new template. The mean squared residual difference between the old and new templates was computed. The mean squared residual difference is defined as in Eq. (1).
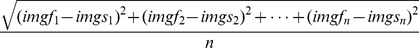
(1)where 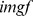 and 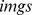 are the initial and the new target images respectively, the subscript *i* is the running voxel position index (ordered as 1, 2, … n as a 1D vector) in the image matrix, and *n* is the total number of voxels. Then the original 24 images were recursively registered to the new average target image. A newer average target image was created from the newly registered images. And the mean squared residual difference was subsequently computed between the current target image and the previous one. This procedure, including registration, averaging and computing the mean squared residual difference, was repeated until the latest mean residual squared difference was stabilized (less than 5% in this current study) [4]. And the latest average target image was our final average image with extracranial tissue.

#### 2.2.2. Creation of canonical brain and intracranial mask

The canonical brain was created by extracting intracranial voxels from the average template image constructed above. We adopted the global histogram threshold method to exclude voxels outside the rat brain. The threshold was determined by Otsu’s criterion [30], which maximizes the between-class variance to get the optimal global threshold value. Prior to determining the threshold, a rectangular bounding box containing the intracranial tissue was defined straightforwardly for each slice with two mouse clicks for the upper left and lower right vertices in MRIcro [25]. And voxels outside the bounding box of the rat brain were removed (assigned zero intensity). Similarly, smaller rectangles over left and right eyes were drawn separately to remove the eyeballs. Finally, the intracranial portion of the image including the olfactory could be identified by collecting the non-zero voxels whose intensities were greater than the global histogram threshold obtained by Otsu’s criterion [30].

Moreover, this extracted canonical brain was processed by binarization transformation to create an intracranial mask image in the standard template space. The origin point of the canonical brain and intracranial mask was the same with the template. Thus, after normalizing to the FDG-PET template, the extracranial tissues of individuals could be automatically removed by this intracranial mask image.

#### 2.2.3. Construction of synthetic PET image

In this study, the paired rat brain structural MRI template and digital atlas in Paxinos & Watson space we constructed before [4] were used for functional effects localization, which comprised from the anterior part of olfactory bulb (z_bregma_ +7.56 mm) to the posterior part of cerebellum (z_bregma_ −15.72 mm). In order to co-register the canonical FDG-PET brain with the MRI structural template and atlas, we created a low-resolution synthetic PET image from the atlas [4]. For this purpose, the 624 regions were merged into six main anatomy structures, all of which could be identified in the FDG-PET template according to the voxel intensity ranges and locations. These 6 anatomy structures included olfactory bulb, cortex, hippocampi, thalamus, mesencephalon and cerebellar. Voxels within each of these 6 regions over the pseudo FDG-PET image were assigned the mean intensity value over the corresponding part in the FDG-PET template. Finally, this synthetic FDG-PET image was further smoothed using an isotropic Gaussian kernel of 2×2×4 mm^3^ FWHM to smooth the juncture of the neighboring segmented parts.

#### 2.2.4. Co-registration of the canonical brain with the atlas in Paxinos & Watson space

The synthetic FDG-PET image was chosen as the target image. The SPM intensity-based affine transformation algorithm and subsequent non-linear warping algorithm was employed to co-register the canonical brain with this target atlas image [31], and the same transformation matrix was applied to the average template image and mask image. At this point, the co-registered template image, canonical brain and mask image were in Paxinos space, which consist the final standard template set. Finally, the origin point of the template set coordinate space was positioned at dorsal 3^rd^ ventricle (D3V).

### 2.3. Construction of the SPM8 plug-ins toolbox named spmratIHEP

In order to analyze rat brain images with less manual manipulation, we constructed a SPM8 plug-ins toolbox named spmratIHEP using MATLAB. Because of the differences between human brain and rat brain, the processing methods and parameter settings in SPM8 must be modified according to the feature of rat brain FDG-PET imaging, which were described in detail as below.

Firstly, spatial normalization is prerequisite in voxel-wise analysis to eliminate individual differences. In order to use the available affine transformation and subsequent non-linear warping algorithm in SPM8, the voxel size of individual brain images was scaled up in the Analyze header by a factor to better approximate human dimensions, which will not affect any interpretation of the statistical results of rat brain.

Then, the constructed FDG-PET template and intracranial mask of rat brain took replacement of the human brain. And the default parameter settings of spatial normalization were adjust according to the feature of rat brain, that the bounding box was adjust to [−150 −180 −126; 150 60 72], the voxel size was adjust to [1 1 1.8] after zooming and the affine regularization was adjust to ‘average sized template’. Moreover, the image matrix of intracranial rat brain images after spatial normalization was sheared to [120 80 98] automatically to cut off the background.

Smoothing was used to improve the signal-to-noise ratio in SPM, in which the Gaussian kernel was adjust to 2×2×4 mm^3^ FWHM, which was approximately two times of the spatial resolution of rat brain after zooming [7].

Finally, in order to display the statistical results of rat brain in Paxinos & Watson space, the definition of coordinates of rat brain took replacement of the human brain. The accurate location of the maximum FDG changes was modified according to the Paxinos & Watson space, in which the x-axis is negative to the left from the midline and positive to the right, the y-axis is positive to the ventral direction relative to the dorsal, and the z-axis is positive to the olfactory bulb direction relative to the bregma and negative to the cerebellum direction. Moreover, a new projection figure of rat brain was created to show the projection of overall blob regions, and a MRI T2-weighted (MRI T2WI) structural image of a single rat brain in Paxinos & Watson space was prepared to show the three-dimensional view of a blob region.

### 2.4. Data analysis of MCAO rat model in the spmratIHEP toolbox

**Figure 1:**
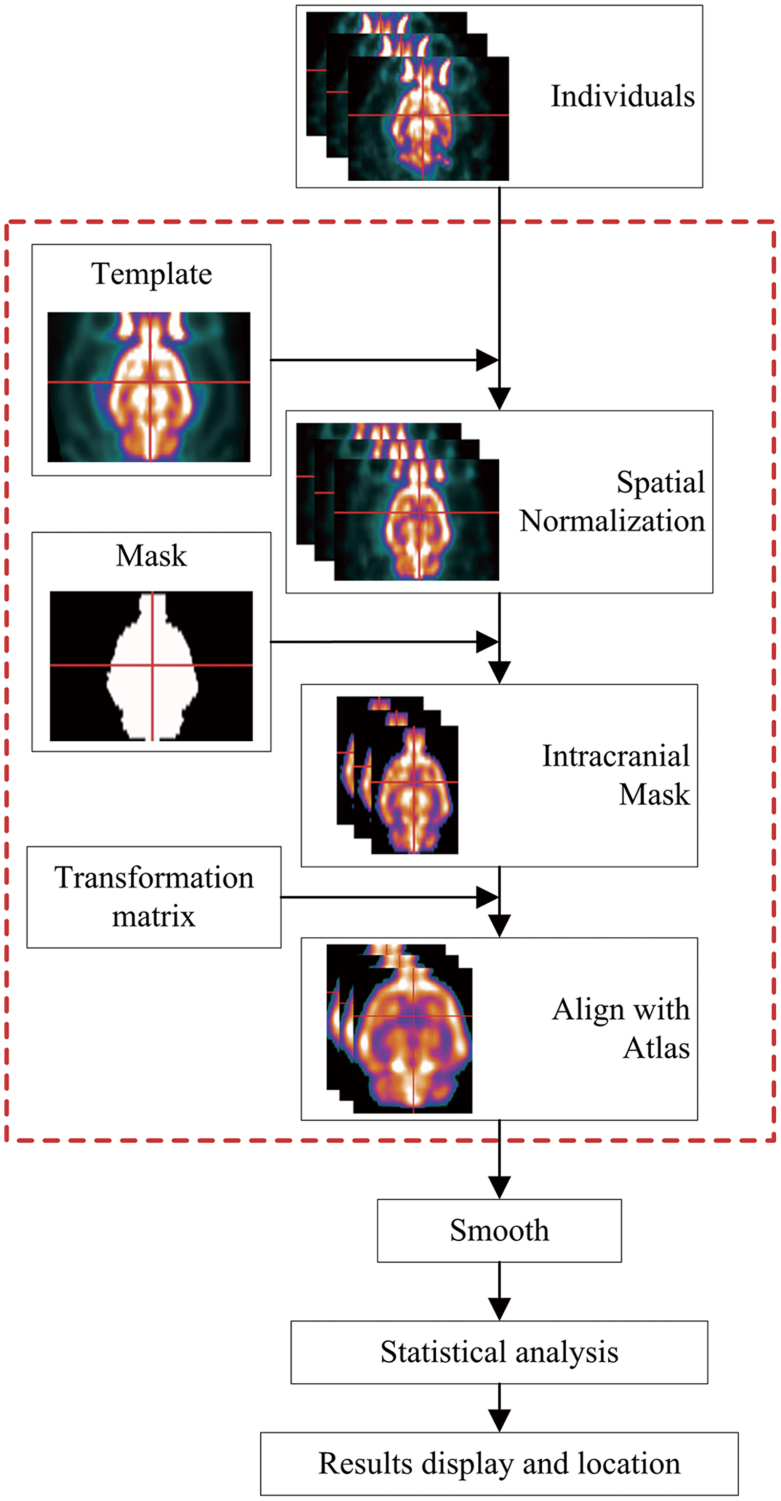
Schematic representation of the data analysis procedure in the spmratIHEP. The procedures of spatial normalization showed in the red dashed pane could be accomplished automatically in this toolbox.

The analysis of MCAO rat model images was performed using our spmratIHEP toolbox to evaluate its practical use. Firstly, the body tissues and background of individual images were manually removed using MRIcro [25] and the origin of the image was repositioned at D3V which is corresponding to the standard FDG-PET template in Paxinos space. Then, the data sets were analyzed in the spmratIHEP automatically as described in the flowchart of Fig. 1. In detail, the FDG-PET images of MCAO rat model and healthy controls were preprocessed as described below. (1) Spatially normalize the individual images of rat brain into Paxinos & Watson space, comprising scaling up the voxel size in the Analyze header by the factor of 4, registering to the FDG-PET template, subsequently removing extracranial tissues via the intracranial image, shearing the matrix to cut off the background. (2) Smooth the normalized images by a Gaussian kernel of 2×2×4 mm^3^ FWHM.

Then, the preprocessed images were analyzed based on the framework of the general linear model (GLM). Two-sample t-test was performed to identify the difference of FDG signals between the rats with MCAO and the healthy controls, in which proportional scaling and intensity normalization was applied to account for global confounds. Finally, the brain regions with significant FDG changes in rats with MCAO were yielded based on a voxel-level height threshold of p<0.05 (FWE corrected) and a cluster-extent threshold of 50 voxels.

## Results

### 3.1. The constructed template set in Paxinos space

**Figure 2:**
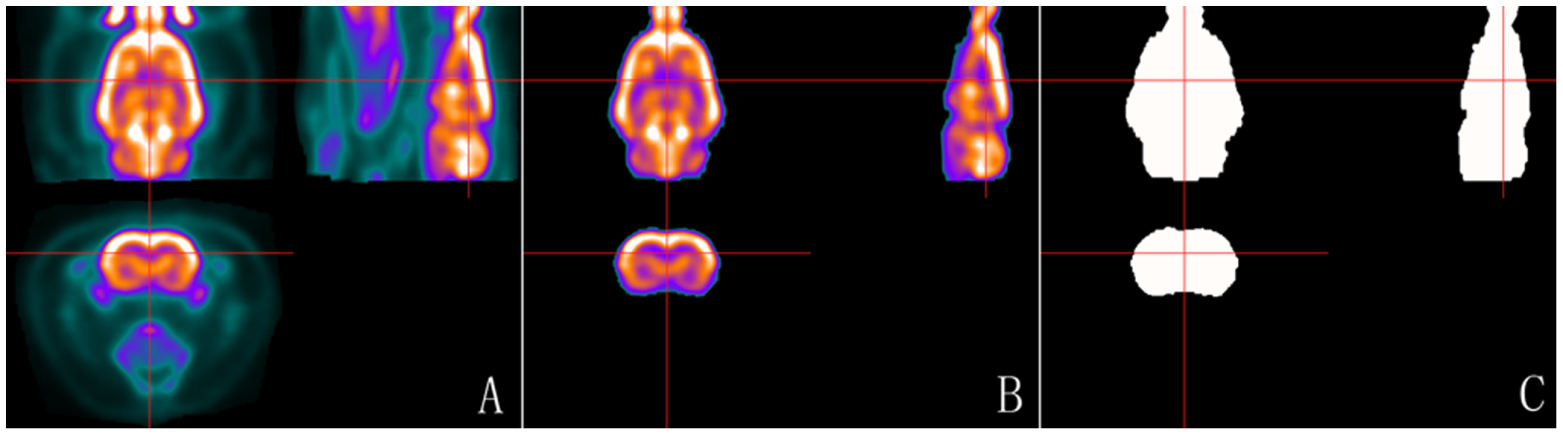
The constructed rat brain template. (A) Axial, sagittal and coronal views of the standard FDG-PET template with extracranial tissues in Paxinos space; (B) axial, sagittal and coronal views of the corresponding FDG-PET canonical brain; and (C) axial, sagittal and coronal views of the corresponding intracranial mask image in Paxinos space. The cross point of red lines represent the origin point D3V. The origin point was the same in the images of template, canonical brain and intracranial mask.

The constructed template set in Paxinos space was shown in Fig. 2, matrix size 300×240×110, voxel size 1×1×1.8 mm^3^ after zooming. This template set was consisted of the standard FDG-PET template with extracranial tissues (Fig. 2A), the corresponding canonical brain (Fig. 2B) and the mask image (Fig. 2C). The original point was on D3V, as the cross point of red lines shown in Fig. 2.

### 3.2. Spatial normalization and extracranial tissue removing of the individual images

**Figure 3:**
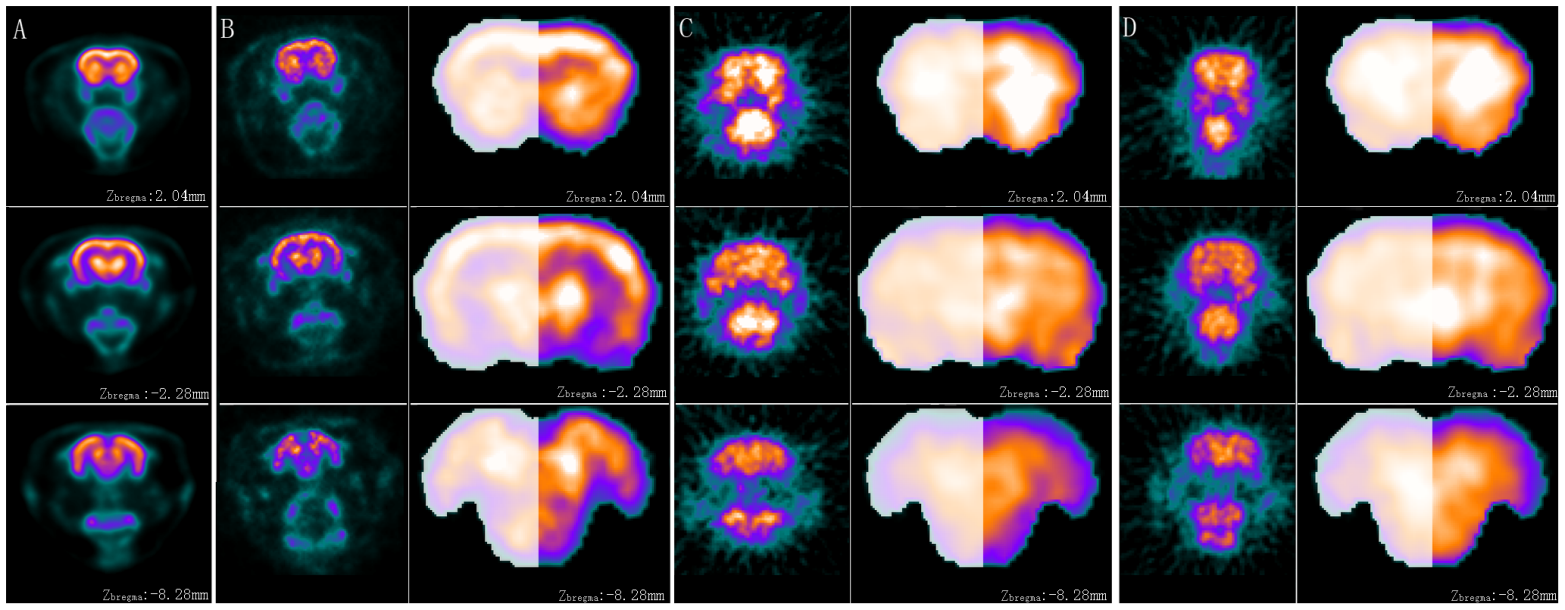
The extraction results. Panel A shows three planes from the standard FDG-PET template of rat brain, whose coordinates were Z_bregma_ 2.04 mm, Z_bregma_ −2.28 mm and Z_bregma_ −8.28 mm separately. Panel B shows the extraction result of a rat which is randomly selected from dataset obtained for template construction. Panel C and Panel D show the extraction results of two rats which are randomly selected for dataset obtained for the intracranial brain extraction evaluations. The original image of individual is shown on the left of Panels B, C, D separately. The extracted intracranial tissue of individual is shown on the right of Panels B, C, D separately, of which the left half shows the intracranial mask image superimposing on the extracted canonical brain and the right half shows the extracted canonical brain. The intracranial mask image is presented as a binary image with 25% transparency, while the extracted canonical brain is presented as a background.

To illustrate the successful use of the intracranial mask image in removing non-brain tissues automatically, both datasets obtained for template construction and spatial normalization evaluations were used. The individual images from both datasets were spatially normalized in spmratIHEP. The extraction of three randomly chosen rats result was shown in Fig. 3, of which one was from the group for template construction (Fig. 3B) and the other two were from the group obtained by E-plus166 system of IHEP (Fig. 3C and Fig. 3D). Moreover, for quantitatively evaluation, three experts, who are the co-authors of this paper (Hua Liu, Xiaofeng Jiang and Baoci Shan), were invited to manually extract the brain tissues from individual FDG-PET images from both two datasets separately by the software ImageJ (Image Processing and Analysis in Java) (http://rsbweb.nih.gov/ij). Then, four volumetric and spatial correspondence measures, Jaccard similarity (JS) [32], [33], the relative error on volume (RV), the proportions of false-positive (FP), and false-negative (FN) [4], [34], [35], were calculated between manually traced out intracranial tissues from three experts, and the manually traced out and automatically extracted intracranial brain volumes. The details of these measures are listed as below.

The Jaccard similarity was defined as in Eq.(2), of which the optimal value is 100%.
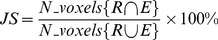
(2)


Where 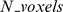 stands for the number of voxels, 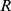 stands for the reference image and 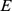 stands for the image to be evaluated.

The relative error on volume was defined as in Eq.(3), of which the optimal value is 0%.
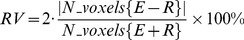
(3)


Where 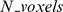 stands for the number of voxels, 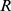 stands for the reference image and 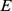 stands for the image to be evaluated.

The proportions of false-positive was defined as in Eq.(4), of which the optimal value is 0%.
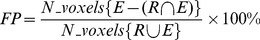
(4)


Where 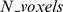 stands for the number of voxels, 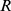 stands for the reference image and 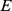 stands for the image to be evaluated.

The proportions of false-negative was defined as in Eq.(5), of which the optimal value is 0%.
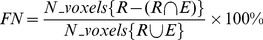
(5)


Where 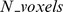 stands for the number of voxels, 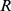 stands for the reference image and 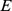 stands for the image to be evaluated.

The calculated results are given in Table 1 and Table 2 separately. As we can see from Table 1 and Table 2, although these two datasets were reconstructed using different reconstruction algorithms and had different spatial resolutions, the intracranial tissue could be extracted successfully by the intracranial mask.

**Table 1:** Volumetric and spatial correspondence measures between manually traced out intracranial tissues from three experts, of which the result is shown as ‘the mean value ± standard deviation’.

	JS (%)	RV (%)	FP (%)	FN (%)
Rat1	91.24±0.94	1.10±0.48	4.38±0.43	4.38±1.09
Rat2	89.77±1.52	6.68±4.60	4.13±3.52	6.10±5.02
Rat3	87.09±2.82	8.93±7.47	6.45±6.31	6.45±6.29

JS (%): Jaccard similarity (the optimal value is 100%);

RV (%): The relative error on volume (the optimal value is 0%);

FP (%): The proportions of false-positive (the optimal value is 0%);

FN (%): The proportions of false-negative (the optimal value is 0%).

**Table 2:** Volumetric and spatial correspondence measures between three manually traced out and automatically extracted intracranial tissues, of which the result is shown as ‘the mean value ± standard deviation’.

	JS (%)	RV (%)	FP (%)	FN (%)
Rat1	91.50±7.36	4.41±3.91	6.31±5.49	2.19±1.92
Rat2	91.74±7.16	1.45±2.04	4.63±4.18	3.63±3.26
Rat3	92.59±6.42	0.10±0.10	3.72±3.22	3.69±3.20

JS (%): Jaccard similarity (the optimal value is 100%);

RV (%): The relative error on volume (the optimal value is 0%);

FP (%): The proportions of false-positive (the optimal value is 0%);

FN (%): The proportions of false-negative (the optimal value is 0%).

### 3.3. Co-registration of the FDG-PET canonical brain to the atlas

**Figure 4:**
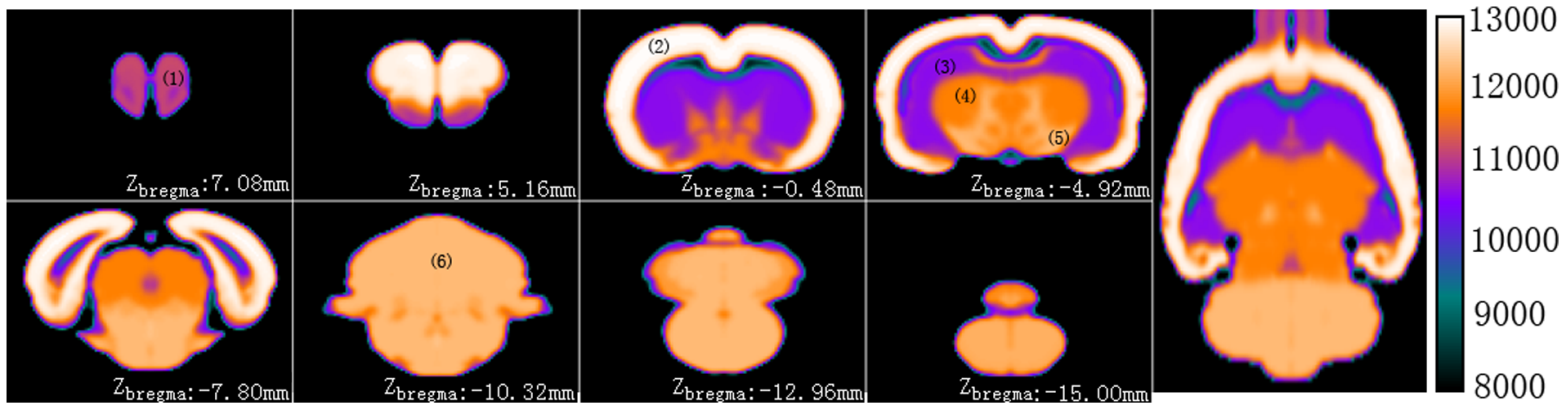
The constructed synthetic FDG-PET images from atlas images in Paxinos & Watson space. It was shown in pseudo-color scaled and the color-bar stands for the intensity of each voxel in synthetic FDG-PET image. The six main anatomy structures were labeled, in which (1) stands for the olfactory bulb, (2) stands for the cortex, (3) stands for the hippocampi, (4) stands for the mesencephalon, (5) stands for the thalamus and (6) stands for the cerebellar.

The strategy we proposed to co-register the FDG-PET rat brain template with the atlas via synthetic FDG-PET image worked well. The pseudo FDG-PET with six major structures is shown in Fig. 4. This image was only used as a target image for co-registering the FDG-PET template.

**Figure 5:**
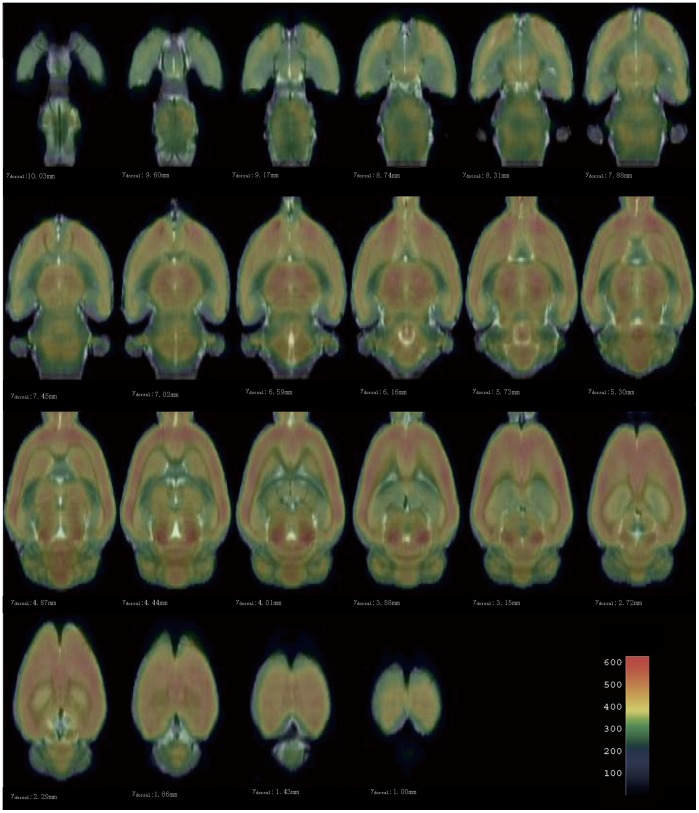
Superimposing the co-registered FDG-PET canonical brain on the MRI T2WI structural canonical brain in Paxinos & Watson space. The co-registered FDG-PET canonical brain is presented with translucency and pseudo-color scaled. The MRI T2WI canonical brain is presented in gray-scale as a background. The color-bar stands for the intensity of each voxel in FDG-PET canonical brain, which is not translucent.

For qualitatively evaluation, the co-registered FDG-PET canonical brain is overlaid on the MRI T2WI structural canonical brain which has already standardized into Paxinos & Watson space in our prior study [4], as shown in Fig. 5. As seen in Fig. 5, the rat brain canonical brain was successfully standardized into the Paxinos space. As illustrated in Figure 5, the FDG-PET canonical brain has been standardized into the atlas space very well.

### 3.4. Data analysis of MCAO rat model in spmratIHEP toolbox

**Figure 6:**
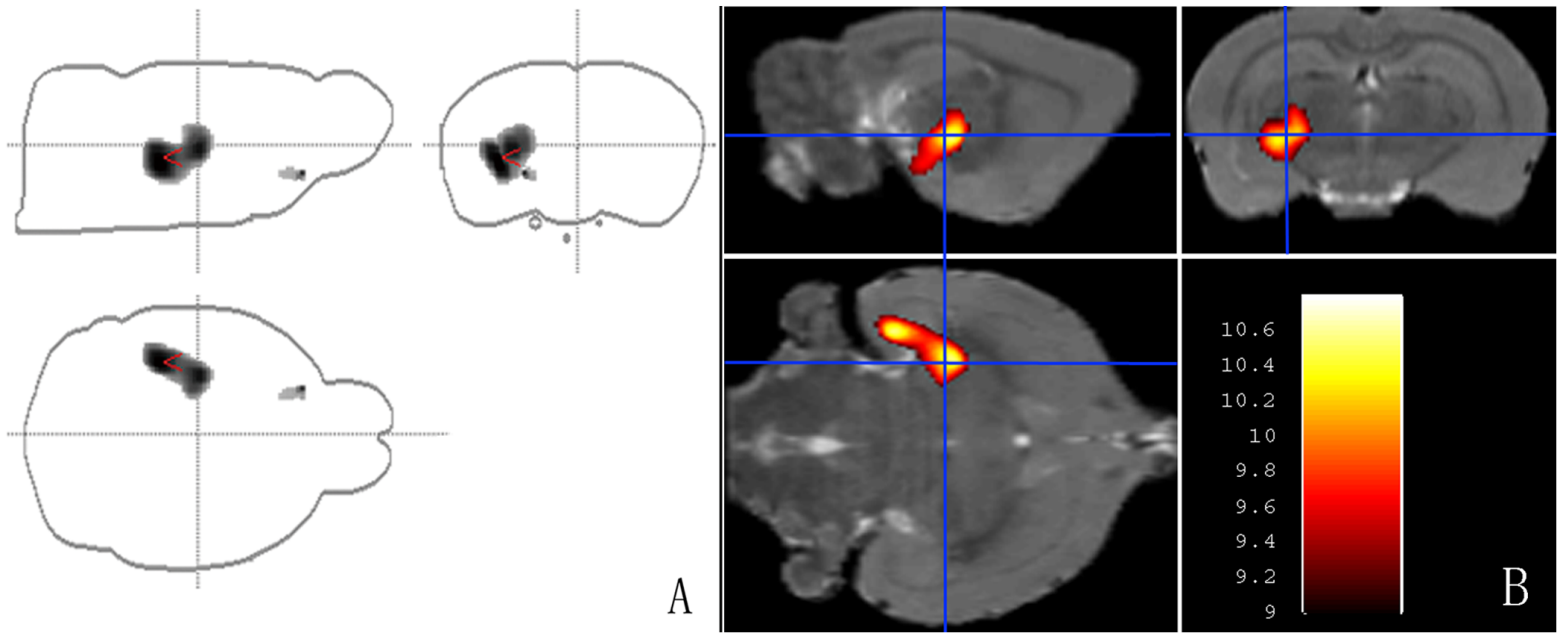
The result of two-sample t-test between the MCAO and healthy controls. (A) The projection of all the blobs were shown in a figure of rat brain, in which the red vees point to the global maximal t-value. (B) The display of the statistical result overlaid on axial, sagittal and coronal views of a structural single brain in Paxinos & Watson space, which is the three-dimensional illustration of one blob. And the color bar stands for the t-value of each significant voxel in Paxinos & Watson space.

The analysis of MCAO rat model images was performed using our spmratIHEP toolbox to evaluate its practical use. The statistical result that brain regions with significant FDG declined in rats with MCAO was shown in a projection figure (Fig. 6A) and fused on structural slices of rat brain in Paxinos & Watson space (Fig. 6B). Meanwhile, the quantitative information of the statistical result was listed in Table 3, in which the ‘Cluster number’ stands for the number of brain regions with significant FDG declined, the ‘K_E_’ stands for the voxel numbers in each cluster, the ‘P_FWE_coor_’ stands for the maximum confidence level in each cluster, the ‘Max_T’ stands for the maximum t-value in each cluster, the ‘Max_Z’ stands for the maximum Z-value in each cluster, the ‘Peak coordinates (mm)’ stands for the coordinates of the maximum point in Paxinos space. These regions are exclusively related to the left side MCA.

**Table 3:** The statistical result of two sample t-test between the MCAO and healthy controls.

Cluster number	K_E_	P_FWE_corr_	Max_T	Max_Z	Peak coordinates (mm)		
					x	y	z
1	2992	0.006	10.79	5.63	−5	7	−7
		0.008	10.55	5.58	−4	5	−4
2	52	0.013	10.14	5.48	−3	6	3

Cluster number: the number of clusters with consecutive voxels with a significant decrease in FDG signal, which is assigned sequentially and artificially. The second line of Cluster 1 which contains large number of contiguous voxels refers to the other significant point in this Cluster.

K_E_: the size of a cluster, in which the number such as 2992 stands for the voxel numbers in the cluster;

P_FWE_corr_: the maximum confidence level in each cluster;

Max_T: the maximum t-value in each cluster;

Max_Z: the maximum Z-value in each cluster;

Peak coordinates (mm): the coordinates of the maximum point in Paxinos & Watson space;

x: the x-axis, which is negative to the left from the midline and positive to the right;

y: the y-axis, which is positive to the ventral direction relative to the dorsal;

z: the z-axis, which is positive to the olfactory bulb direction relative to the bregma and negative to the cerebellum direction.

## Discussion

In the current study, we established a SPM8 plug-ins toolbox named spmratIHEP for voxel-wise analysis of FDG-PET images of rat brain, in which an FDG-PET template and an intracranial mask image of rat brain in Paxinos & Watson space [4], [18] were constructed, and the default settings were modified according to the feature of rat brain.

For voxel-based statistical analysis of functional images, all the individual images should be primarily normalized into one common space, such as the MNI (Montreal Neurological Institute) space in human brain studies. And the standard brain template performs as a general criterion in spatial normalization. In our study, to spatially normalizing individual rat brain images, an FDG-PET rat brain template was iteratively created from 24 healthy adult rats. Because there are minor differences in anatomical structures between healthy ones [7], our created FDG-PET rat brain template could be a group representation of SD rat brain. Furthermore, because there is a close association between olfactory dysfunction and cognitive impairment [14]–[16], our constructed FDG-PET template comprised not only the cerebrum and cerebellum, but also the whole olfactory bulb, which made the later cognitive studies much more exhaustive.

How to locate the anatomical regions of functional effects in statistical results is important in voxel-wise analysis. In physiological significance location, the brain atlas also called stereotaxic coordinates corresponding to the template space provides detailed anatomical information for functional results, such as the Tailarach coordinates for human brain studies [17]. In this study, one of the most popular atlases in rat brain studies, the stereotaxic coordinates established by Paxinos & Watson [18], was used for functional effects location, based on which our group has constructed the digital atlas and corresponding MRI T2WI structural canonical brain in Paxinos & Watson space [4]. The technique of synthetic FDG-PET was employed [10], [19] to standardize the FDG-PET canonical brain into the Paxinos & Watson space. As illustrated in the Result part, the FDG-PET canonical brain was standardized into the atlas space very well. Although the anatomical structures are excellently delineated in Paxinos and Watson [18], the accuracy of anatomical coordinates for statistical result was millimeter because of the low resolution of FDG-PET imaging.

In addition, to evaluate the performance of spmratIHEP in voxel-wise analysis of FDG-PET images, nine rats with left side MCAO and eight healthy controls, acquired on Siemens Inveon PET which differs from the one imaging for template construction, were enrolled in this study. The data preprocessing and voxel-wise analyzing of these two groups were all performed automatically in spmratIHEP based on SPM8. As illustrated in the Result part, the statistical result was almost related to the left side MCA. Therefore, our constructed toolbox performed well in rat brain imaging data analyzing in SPM8.

In conclusion, demonstrating its adequacy and practical usage in SPM environment, we reported the constructed FDG-PET rat brain template in the stereotaxic coordinate space of the Paxinos and Watson rat brain atlas. We believe it will be helpful to streamline the neuroimaging data analyses for rat brain images from the pre-processing stage to the reports of the statistical inference results.
